# Impact of close-proximity air pollution on lung function in schoolchildren in the French West Indies

**DOI:** 10.1186/s12889-015-1382-5

**Published:** 2015-01-31

**Authors:** Brice Amadeo, Céline Robert, Virginie Rondeau, Marie-Alice Mounouchy, Lucie Cordeau, Xavier Birembaux, Eddy Citadelle, Jacques Gotin, Monique Gouranton, Gérard Marcin, David Laurac, Chantal Raherison

**Affiliations:** ISPED, Centre INSERM U897-Epidemiologie-Biostatistique, Univ. Bordeaux, F-33000 Bordeaux, France; INSERM, ISPED, Centre INSERM U897-Epidemiologie-Biostatistique, F-33000 Bordeaux, France; Association Karu-Asthme, F-97110 Pointe-à-Pitre, Guadeloupe; CHU Bordeaux, service de Pneumologie, F-33000 Bordeaux, France; ISPED, Equipe Santé Travail Environnement, Université de Bordeaux, 146 rue Léo Saignat, 33076 Bordeaux, France

**Keywords:** Asthma, ISAAC, Peak expiratory flow, Pollutants, Schoolchildren

## Abstract

**Background:**

High levels of asthma prevalence and severity of respiratory symptoms have been found in the Caribbean but little is known about the impact of air pollution in these regions.

This study aimed to describe air pollution and measure the associations with child lung function in Guadeloupe (French West Indies).

**Methods:**

Data from 30 randomly chosen elementary schools (8–13 years old) were obtained using a standardized protocol adapted from the ISAAC2 study. We considered two health outcomes: peak expiratory flow (PEF) before running and the variation in peak expiratory flow (ΔPEF) after running. The associations between pollutants and outcomes were investigated using several air pollution exposure models: i) medium-term exposure to close-proximity pollution both indoor and outdoor for ozone (O_3_) and nitrogen dioxide (NO_2_) and ii) short- and medium-term exposure to background pollution for O_3_, NO_2_, sulphur dioxide (SO_2_) and small particulate matter (PM_10_).

**Results:**

Of 1,463 children, 277 (16%) were found to have asthma. A 1-μg/m3 increase in medium-term exposure to outdoor close-proximity pollution by O_3_ was associated with a PEF decrease (β = −0.32; 95% CI: −0.61;-0.03). No association was found with NO_2_ regarding close-proximity pollution. The association between medium-term exposure to background pollution and PEF decrease was stronger in asthmatic children than in non-asthmatic children for O_3._ No reduction in PEF or ΔPEF was shown with NO_2_, SO_2_ and PM_10_ pollutants but a significant association was found between PM_10_ and PEF increase.

**Conclusions:**

Our results suggest that O_3_ could have an acute effect on child lung function in the Caribbean even at a low concentration (below the WHO guidelines). Further research in the Caribbean is needed to confirm these findings.

## Background

As with obesity and diabetes, the prevalence of asthma and allergic diseases has dramatically increased during the last three decades, particularly in children [1]. The possible explanations for this worldwide increase are either genetic variability or changes in environmental factor exposure. The genetic predisposition of each individual has been shown to play a role in the development of allergic diseases, especially in the pathophysiological processes involved. With current knowledge of population genetics, a recent modification in genetic variability seems unlikely. However, the assumption of an interaction between individual susceptibility and environmental stimuli after birth is possible. This interaction could produce epigenetics that subsequently influence susceptibility to chronic inflammatory disease [2]. Shifts in environmental factors including exposure to air pollutants, allergens and infections might be associated with this increase.

Several epidemiological studies have investigated the associations between exposure to urban air pollution and respiratory and allergic diseases among children, and have identified positive associations between background concentrations of nitrogen dioxide (NO_2_) [3,4], sulphur dioxide (SO_2_) [5,6], particulate matter (PM) [7,8] and ozone (O_3_) [9,10].

Particles and NO_2_ have shown more significant results. Negative associations between air pollution and pulmonary health were more pronounced in asthmatic children in this review [11]. Peak expiratory flow (PEF) was the most usual measurement for children’s lung function, followed by the measure of forced expiratory volume in one second (FEV1). In a meta-analysis, Weinmayr et al. reported for 51 studies (36 from Europe and 15 elsewhere mainly in USA) short-term effects of PM_10_ and PM_10_ on respiratory symptoms and lung function mainly PEF [7]. For an increase of 10 μg/m^3^ of PM_10_, they found a significant increase of 2.8% in asthma symptoms, an increase for cough (1.2%), and a decrease of PEF (−0.082 l/min). In addition, a higher decrease of PEF was found in asthmatic (−0.549 l/min) compared with others children (0.010 l/min). However, the impact of ozone on respiratory health was not studied in this meta-analysis. In healthy children living in UK, an increase of 1.0 μm^2^ in the mean arear of black carbon in airway macrophage, was associated with a decrease of 17% in FEV1 [12]. In USA, a longitudinal study of children in 12 communities in California, reported a significant correlation between reduced lung function growth and the background concentration in PM_10_ [13]. In Brazilia, Jacobson et al. in a panel study estimated the effect of current levels of outdoor pollution on peak expiratory flow [14]. The global effect was important for PM_10_ with PEF reduction of 0.31 l/min (CI 95%: −0.56;-0.05), however, ozone was not studied in this analysis. More recently, a nationwide in Taiwan, was conducted on schoolchildren age 6–15 years (n = 1494) and showed that subchronic exposure to ambient PM_2.5_ and ozone leads to reduce lung capacity, and acute exposure to ozone decreases mid-expiratory flow (−0,123 l) [15].

The impact of background air pollution on respiratory diseases seems to vary in different parts of the world probably due to differences in the spatial and temporal variability of the composition and sources of air pollutants between different urban areas [16]. To our knowledge, little is known about the impact of air pollution in the Caribbean with its tropical wet and dry environments (defined by relatively constant temperatures throughout the year and seasonal variations dominated by rainfall), while levels of asthma prevalence and severe respiratory symptoms in children are mostly higher than in Northern Temperate Zones [17].

Population-based surveys of Caribbean children have reported that over 13% of participants admitted to having a past or present diagnosis of asthma [18-20]. In Guadeloupe, which is a French West Indies department located in the Caribbean, previous studies also reported high asthma prevalence in children under 13 years old and a hospital admission risk related to asthma 1.9-fold higher than in Metropolitan France [19,21].

The present study aimed to provide data on air pollution exposure in Guadeloupe and to measure the associations between air pollution and lung function in elementary schoolchildren. The associations between the pollutants and lung function were assessed using several air pollution exposure models: i) medium-term exposure to close-proximity pollution both indoor and outdoor and ii) short- and medium-term exposure to background pollution.

## Methods

### Participants and study design

**Figure 1:**
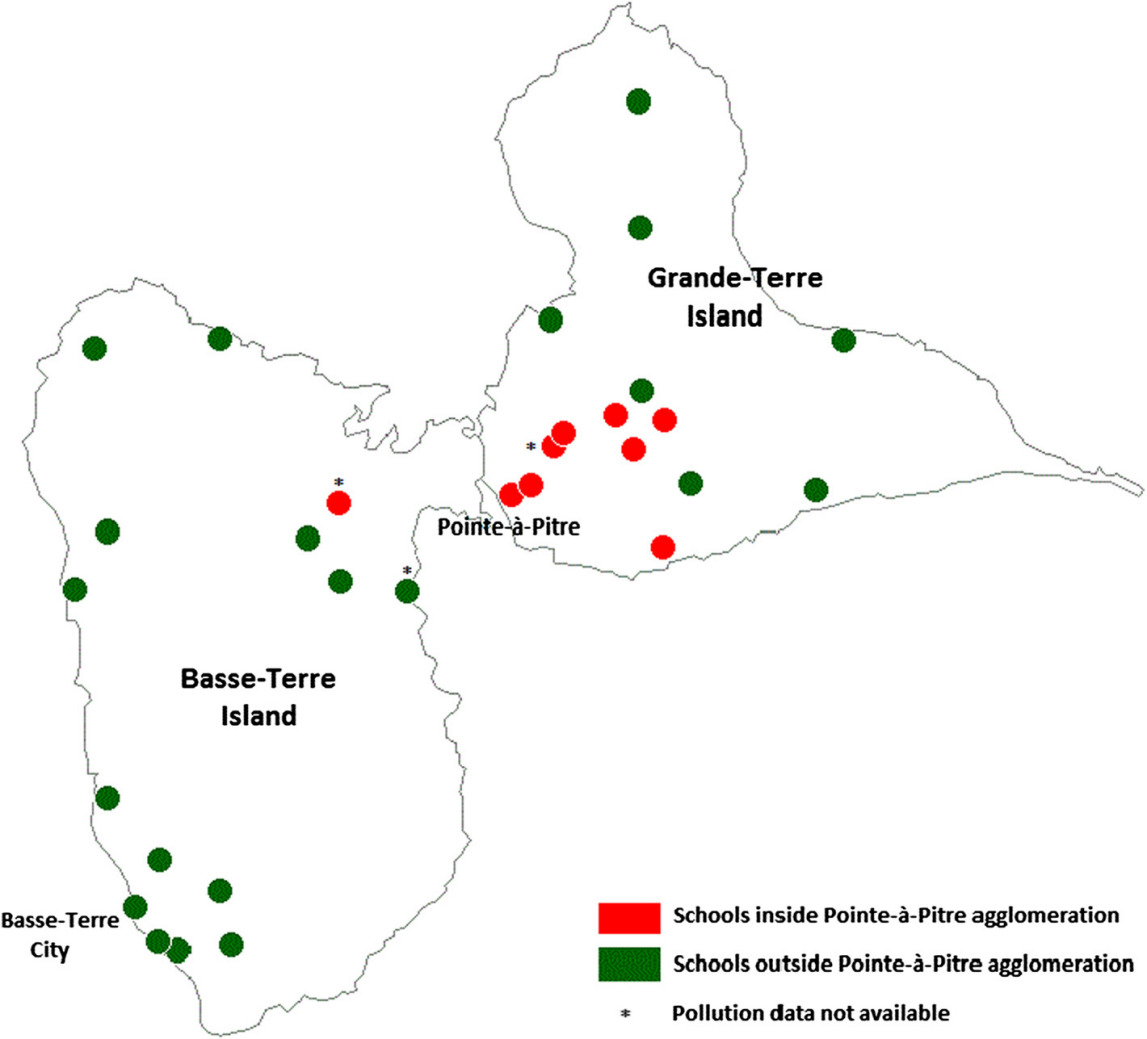
**Map of Guadeloupe and localization of schools participating in the ISAAC 2 Guadeloupe study.** Note. This map was made with Philcarto® software.

This cross-sectional study was carried out between December 2008 and December 2009 using the second phase of the International Study of Asthma and Allergy in Childhood (ISAAC II) standardized protocol, which has been widely described in the literature [22]. A random representative sample of 30 elementary schools in Guadeloupe was chosen from a complete list of all elementary schools (Figure 1). As specified in the ISAAC II protocol [22], the minimum required sample size had to be above 1,000 children. Among the 30 elementary schools, completed valid data on clinical examination, questionnaire and pollution were available in 27 schools representing 1,436 children distributed in 91 classrooms. In three schools, pollution data could not be collected owing to a long strike in Guadeloupe (Figure 1).

The questionnaire contained the core ISAAC questions on respiratory and allergic diseases, how these where managed and potential risk factors. The clinical examination included the standardized protocol of the running test to assess exercise-induced asthma that was previously used in the French Six-City Study [8]. The detailed method has been described elsewhere [23]. The research protocol was approved by the National Ethics Board and all children and parents gave written informed consent. The timetable of the school visit for air pollution exposure assessment and the clinical examination of the children were randomly chosen.

During the study period, lung function using peak expiratory flow (PEF) was measured for each subject. Both background exposure and proximity exposure were used to assess personal exposure. Proximity pollution is an accurate measure of local pollution exposure. However, in our study, we only had the possibility to measure a part of the proximity pollution to which children were exposed (only at school) owing to economic and organizational constraints. For this reason, measuring background exposure was useful in our study.

Regarding medium-term exposure, concentrations of O_3_ and NO_2_ for close-proximity air pollution and concentrations of O_3_, NO_2_, and SO_2_ and particulate matter with a diameter below to 10 μm (PM_10_) for background air pollution were estimated for each classroom. All children in a classroom were assigned the same estimated pollution exposure. Regarding short-term exposure, the different lags of pollutant levels, corresponding to the measurement of pollutant levels the current day up to five days (D0, D1, D2, D3, D4, D5) before the measurement of PEF were estimated for each child.

### Guadeloupe

Guadeloupe, a French overseas department located in the Caribbean (Figure 1), comprises two main islands: Basse-Terre Island and Grand-Terre, which are separated by a narrow sea channel. The land area represents 1,628 square kilometers with a density population of 249 inhabitants per square kilometers. The most important two cities are Basse-Terre, which is the capital of Guadeloupe, and Pointe-à-Pitre. Basse-Terre Island has a rugged terrain due to volcanic mountains, which explains why the main road network is only developed in Grande-Terre Island and around these two cities.

### Health outcomes

Two health outcomes were investigated using the run test: baseline peak expiratory flow (PEF) before running and variation in peak expiratory flow (ΔPEF) after running. Baseline PEF was measured three times immediately before the run test with a Mini-Wright peak flow meter in accordance with the American Thoracic Society guidelines [24]. The maximum of these measures was retained and compared to the predicted value. The predicted value was assessed using the height value and the abacus built through the curve of Godfrey [25]. All the children with PEF superior or equal to 70% of the predicted value before running were invited to undergo a 6-min period of running. PEF was measured 5 and 10 min following the run test. The maximum of this second series of measurements was used to calculate the ΔPEF corresponding to the percentage decrease in PEF after exercise compared to the baseline PEF. Measurements of PEF were performed by a spirometry technician after training and a senior pulmonologist supervised the procedure. The interest of measuring the peak expiratory flow before running and the peak expiratory variation after it was to determine whether pollution had any impact on breathing at rest or on breathing after exercise, respectively. In addition, ΔPEF represents exercise-induced asthma, which is considered to be a distinct asthma phenotype. Therefore, we exclude children with a PEF lower than 70% of the predicted value, i.e. probably the most vulnerable children.

The following allergic manifestations were considered: asthma was defined by an affirmative response to the question ‘Has your child ever had asthma?’; atopic dermatitis by an affirmative response to the question ‘Has your child ever had itchy rash affecting any of the following places: the folds of the elbows, behind the knees, in front of the ankles, under the buttocks, or around the neck, ears or eyes?’; and atopy by an affirmative response to at least one of the three questions ‘Has your child ever had eczema?’ or ‘Has your child ever had hay fever?’ or ‘Has your child ever had another form of allergic rhinitis other than hay fever, i.e. caused by allergens other than pollens?’. In this study, we chose to analyze lifetime asthma in order to have a more specific diagnosis of asthma for children. The question on wheezing during the 12 past months is more sensitive than specific.

### Assessment of exposure to air pollution

Data regarding medium-term exposure to close-proximity and background air pollution were provided by the Air Quality Monitoring Networks of Guadeloupe (GWAD’AIR Association) for a period of two consecutive weeks before the day the children received their clinical examination.

Medium-term exposure to close-proximity pollution by O_3_ and NO_2_ was measured in 27 participating schools. Levels of each pollutant were assessed using passive diffusion samplers in representative points of each classroom for indoor air pollution and near the playground for outdoor air pollution. The method has already been described elsewhere [26].

With regard to indoor pollution, the data showed there was little variation between classrooms in the same school. For this reason, the concentration average per school was used (i.e. 27 data points per pollutant). Background air pollution was measured using fixed stations located in three cities (Pointe-à-Pitre, Abymes and Baie-Mahault). According to the GWAD’AIR Association, background air pollution can be considered as uniform in all schools located in the agglomeration of Pointe-à-Pitre i.e. 7 schools recording pollution data (Figure 1).

The close-proximity exposure in the 27 schools reflected both spatial and temporal variations since schools were sample at different points in time and space. For background pollution, the pollutant concentration was considered as the same in the area where the 7 schools were located. For this reason, the background exposure in the 7 schools represented only temporal variations.

The daily concentration of each air pollutant was calculated from the maximum daily 1-hour average for O3, NO2 and SO2 and the daily average for PM10 according to European guidelines (http://www.airqualitynow.eu). Medium-term exposure to background air pollution was defined by the average daily concentration for the two weeks before the clinical examination. Short-term background air pollution was defined by the daily average concentration of each pollutant on the clinical examination day and up to the five subsequent days corresponding to five different lags (D0, D1, D2, D3, D4, D5). These lags were classified into two categories: short delay exposure and cumulative short delay exposure, which indicates the mean exposure to pollutants in the five days before.

In summary, two analyses were performed: a first analysis regarding close-proximity pollution in 27 schools and a second analysis regarding background pollution in a restricted sample of 7 schools (Pointe-à-Pitre agglomeration). The variation in estimated pollution data could be both spatial and temporal in the first analysis whereas in the second analysis, it could only be temporal.

Particulate matter with a diameter inferior to 2.5 μm (PM2.5) was not considered in this study because it was not monitored by the GWAD’AIR Association when the study started.

### Statistical analysis

Data were analyzed using the statistical package SAS (Version 9.1; SAS Institute Inc., Cary, NC, USA). We built several linear mixed regressions to measure the associations between lung function (PEF and ΔPEF) and medium-term exposure to i) close-proximity pollution (indoor and outdoor) by O_3_ and NO_2_ and ii) background pollution by O_3_, NO_2_, SO_2_ and PM_10_. With respect to short-term exposure to background air pollution, we tested the associations between the lagged pollutant levels, defined previously, and lung function using a distributed-lag model. This approach reduces collinearity, decreases the number of parameters to be estimated via the polynomial function and allows estimation of specific lagged effects [27].

We fitted separate models for each pollutant. Variables such as sex, age, BMI, full-term birth, rainy season, temperature and relative humidity were considered as confounding factors and were forced in all models. The other explanatory variables were retained in multivariable models if the associations with the outcomes were statically significant or if they were a confounding factor. The interactions with “asthma” or “drugs to treat asthma before clinical examination” variables were tested in each model in order to check for a potential modification effect with the pollutants. Children were not independent observational units because they were nested within schools. For this reason, a random intercept was introduced in each model in order to take into account the dependence in the outcome for children in the same school.

To summarize, the linear mixed model is defined as follows:

$$ {Y}_{ci}={\beta}_0+{\gamma}_{0c}+{\beta}_1\times Pollutan{t}_{ic}+\beta \times {X}_{ic}+{\varepsilon}_{ic} $$

where *Y*_*ci*_ is the dependent variable (PEF or ΔPEF) with *c* which is the elementary school (c = 1,…,k) and *i* a child in an elementary school. *β*_0_ is the fixed intercept and *γ*_0*c*_ is the random intercept at school level. *β*_1_ is the fixed pollutant effect and *Pollutant*_*ic*_ is the average medium-term pollutant concentration for child *i* in school *c. β* is the coefficient vector of fixed effects for adjustment factors and *X*_*ic*_ is the variable matrix for the adjustment factors. The quantities *ε*_*ic*_ are random variables representing errors in the relationship.

The distributed-lag model to study short-term exposure to pollution is as follows:

$$ {Y}_{cit}={\beta}_0+{\gamma}_{0c}+{\zeta}^T\times {X}_{ct}+{\xi}^T\times {X}_{ic}+{\displaystyle \sum_{l=0}^5}{\delta}_l\times \left({Z}_{ic,t-l}\right)+{\varepsilon}_{ic} $$

where t is the clinical examination day, *ζ*^*T*^ is the transpose vector of fixed effects for time-dependent variables (e.g. rainy season) with *X*_*ct*_ the matrix of these variables, and *ξ*^*T*^ is the transpose vector of fixed effects for individual variables with *X*_*ic*_ the matrix of these variables. Pollutant effects were taken into account using ${\displaystyle \sum_{l=0}^5}{\delta}_l\times \left({Z}_{ic,t-l}\right),$ where pollutant effects that were delayed by up to five days and *δ*_*l*_ was constrained to follow a polynomial function of degree 2. *Z*_*ic*,*t* − *l*_ is the pollutant concentration for day *t* at lag *l* for a child (*i*) in an elementary school (*c*).

## Results

### Children’s characteristics

Children characteristics are presented in Table 1. Among the 1,436 children, the average age was 10.3 years (SD **±** 0.8; range: 8.2-12.9), 46.5% were males and 21.3% had a high BMI. Regarding their family situation, 52.4% of children lived with both parents. The majority of parents had a primary or secondary educational level (63.5%). With respect to environmental factors, the relative humidity and temperature were 68.6% (SD **±** 15.3; range: 25–95) and 27.8°C (SD **±** 1.9; range: 22–33) respectively. Data were collected during the rainy season in 44.5% of children. Exposure to tobacco smoke concerned 14.3% of children. The prevalence rates of asthma, atopy, atopic dermatitis, drugs used to treat asthma before clinical examination, family history of allergy and premature birth were 15.5%, 24.5%, 2.4%, 5.2%, 24.3% and 27.2% respectively. The values of PEF and ΔPEF were on average 272 L/min (SD **±** 47.8; range: 130–460) and minus 1% (SD **±** 9.9; range: −56%-97%) respectively. Mean height in the sample of children was 146.0 cm (SD **±** 9.4).

**Table 1:** **Children from the 27 participating schools and the 7 schools in the Pointe-à-Pitre agglomeration**

**Variables**	**Participating schools including 1,436 children**		**Participating schools in Pointe-à-Pitre agglomeration including 506 children**	
	**% data provided**	**Mean ± SD or Frequency**	**% data provided**	**Mean ± SD or Frequency**
**Socio-demographic factors**				
Age	100.0	10.3 ± 0.8	100.0	10.5 ± 0.7
Sex (male)	100.0	46.5	100.0	47.4
BMI	99.2		98.6	
*Low*		6.7		7.4
*Normal*		72.0		70.8
*High*		21.3		21.8
**Family situation of children**	88.6		88.3	
*with both parents*		52.4		48.5
*with mother*		43.4		47.9
*with father*		2.0		2.7
*Other*		2.2		0.9
**Educational level of parents**	76.9		76.1	
*Primary or secondary*		63.5		64.2
*High school and university*		36.5		35.8
**Environnemental factors**				
Relative humidity (unit: %)	100.0	68.6 ± 15.3	100.0	59.6 ± 15.2
Outside temperature (unit: °C)	100.0	27.8 ± 1.9	100.0	28.1 ± 1.4
Rainy saison (%)	100.0	44.5	100.0	35.4
Exposure to tobacco smoke (%)	57.9	14.3	61.7	12.2
**Medical history**				
Asthma (%)	100.0	15.5	100.0	16.6
Atopy (%)	100.0	24.5	100.0	23.7
Atopic dermatitis (%)	100.0	2.4	100.0	3.6
Drugs against asthma before clinical examination (%)	100.0	5.2	100.0	7.1
Family history of allergy (%)	100.0	24.3	100.0	25.5
Premature birth (%)	81.8	27.2	91.3	31.8
**Lung function**				
Peak expiratory flow before run (unit: L/min)	97.1	271.5 ± 47.8	96.8	274.2 ± 49.9
Variation of peak expiratory flow after run (unit: %)	80.6	−1.1 ± 9.9	78.7	−0.5 ± 12.0

Abbreviations: SD: standard deviation; BMI: Body Mass Index.

Educational level of parents and exposure to tobacco smoke had a high number of missing data (23.1% and 42.1%, respectively). For this reason, the latter were not taken into account in the models.

### Exposure to air pollutants

Table 2 summarizes the distribution of air pollutant concentrations estimated for medium-term exposure to close-proximity air pollution (indoor and outdoor) and background air pollution.

**Table 2:** **Concentration of medium-term air pollutants in schools participating in the study**

**Pollutants**	**WHO air quality guidelines 2005**	**Type of pollution**	**Number of schools**	**in μg/m** ^**3**^			
				**Mean**	**Standard deviation**	**Minimum**	**Maximum**
**O**_**3**_	100 μg/m^3^ 8-hour mean	Indoor close-proximity	27	49.5	14.8	23.6	80.1
		Outdoor close-proximity	27	55.3	16.4	21.1	90.9
		Background	7	54.1	6.8	40.5	59.2
**NO**_**2**_	40 μg/m^3^ annual mean	Indoor close-proximity	27	5.3	4.3	0.9	21.5
		Outdoor close-proximity	27	5.3	3.5	0.9	15.3
		Background	7	14.8	3.8	11.5	22.1
**SO**_**2**_	20 μg/m^3^ 24-hour mean	Background	7	4.7	3.7	1.8	12.7
**PM**_**10**_	20 μg/m^3^ annual mean	Background	7	23.9	6.8	16.5	33.4

Abbreviations: O_3,_ ozone; NO_2_, nitrogen dioxide; SO_2_, sulfur dioxide; PM_10_, particles with aerodynamic diameter lower than 10 μm.

The mean medium-term exposure to indoor close-proximity air pollution was 49.5 μg/m^**3**^ (SD **±** 14.8 μg/m^**3**^) for O_3_ and 5.3 μg/m^**3**^ (SD **±** 4.3 μg/m^**3**^) for NO_2_, while the mean medium-term exposure to outdoor close-proximity air pollution was 55.3 μg/m^**3**^ (SD **±** 16.4 μg/m^**3**^) for O_3_ and 5.3 μg/m^**3**^ (SD **±** 3.5 μg/m^**3**^) for NO_2_. Wide minimum-maximum intervals were found between the 27 participating schools (e.g. for indoor, O_3_ range: 23.6-80.1 μg/m^**3**^ and NO_2_ range: 0.9-21.5 μg/m^**3**^). A high correlation between indoor and outdoor close-proximity air pollution was identified with a Spearman’s correlation coefficient of 0.68 for O_3_ and 0.88 for NO_2_.

Data regarding medium-term background air pollution restricted to schools in the Pointe-à-Pitre agglomeration were 54.1 μg/m^**3**^ (SD **±** 6.8 μg/m^**3**^) for O_3,_ 14.8 μg/m^**3**^ (SD **±** 3.8 μg/m^**3**^) for NO_2,_ 4.7 μg/m^**3**^ (SD **±** 3.7 μg/m^**3**^) for SO_2_ and 23.9 μg/m^**3**^ (SD **±** 6.8 μg/m^**3**^) for PM_10._ There was no meaningful correlation between close-proximity and medium-term background air pollution.

Pollutant concentrations were lower than WHO guidelines for O_3_, NO_2_ and SO_2_. However, five of the seven schools (i.e. 352 children) were exposed to more than 20 μg/m^**3**^ of PM_10_ i.e. above WHO guidelines.

### Associations between medium-term exposure to close-proximity pollution and health outcomes

Tables 3 and 4 show coefficients and 95% CI of PEF and ΔPEF outcomes associated with the 1-unit change of mean of each pollutant.

**Table 3:** **Results of linear mixed models to investigate associations between medium-term exposure to indoor and outdoor close-proximity air pollution and peak expiratory flow (n = 1133)**

	**Indoor**				**Outdoor**			
	**O**_**3**_ **(in μg/m** ^**3**^ **)**		**NO**_**2**_ **(in μg/m** ^**3**^ **)**		**O**_**3**_ **(in μg/m** ^**3**^ **)**		**NO**_**2**_ **(in μg/m** ^**3**^ **)**	
	**β**	**(95% CI)**	**β**	**(95% CI)**	**β**	**(95% CI)**	**β**	**(95% CI)**
Pollutant	−0.21	(−0.55; 0.13)	0.15	(−0.85; 1.16)	**−0.32**	(−0.61; −0.03)	−0.04	(−1.25; 1.17)
Sex (male vs girl)	**5.73**	(0.50; 10.96)	**5.59**	(0.36; 10.81)	**5.75**	(0.53; 10.97)	**5.57**	(0.35; 10.80)
Age	**22.17**	(18.58; 25.76)	**22.03**	(18.44; 25.61)	**22.01**	(18.43; 25.58)	**22.04**	(18.45; 25.62)
BMI								
*High vs Normal*	**19.13**	(12.74; 25.52)	**19.01**	(12.62; 25.41)	**19.03**	(12.66; 25.4)	**18.97**	(12.58; 25.36)
*Low vs Normal*	**−18.60**	(−28.58; −8.63)	**−18.33**	(−28.30; −8.36)	**−18.78**	(−28.74; −8.82)	**−18.29**	(−28.26; −8.32)
Premature birth (Yes vs No)	−5.32	(−11.19; 0.55)	−5.22	(−11.09; 0.65)	−5.35	(−11.21; 0.52)	−5.23	(−11.10; 0.64)
Rainy season (Yes vs No)	0.14	(−8.41; 8.70)	−0.85	(−10.23; 8.53)	1.64	(−6.24; 9.53)	−0.25	(−9.42; 8.92)
Outside temperature (unit: °C)	1.82	(−0.05; 3.68)	**2.10**	(0.28; 3.92)	**1.85**	(0.09; 3.61)	**2.10**	(0.29; 3.92)
Relative humidity (unit: %)	0.27	(−0.08; 0.61)	0.26	(−0.12; 0.65)	0.19	(−0.15; 0.54)	0.29	(−0.08; 0.67)
Atopy (Yes vs No)	**7.53**	(1.44; 13.62)	**7.50**	(1.41; 13.60)	**7.42**	(1.33; 13.50)	**7.52**	(1.42; 13.61)
Asthma (Yes vs No)	**−9.62**	(−16.85; −2.39)	**−9.60**	(−16.83; −2.37)	**−9.55**	(−16.77; −2.33)	**−9.63**	(−16.86; −2.40)
Pointoise agglomeration (Yes vs No)	7.82	(−3.00; 18.64)	4.89	(−7.99; 17.76)	7.59	(−1.87; 17.06)	6.17	(−6.29; 18.62)
Variability of school random intercept: ${\widehat{\upsigma}}_{\upgamma 0}^2$ (*P* value)	**43.39 (0.02)**		**48.33 (0.01)**		28.43 (0.09)		**47.91 (0.01)**	

Note. The peak expiratory flow (PEF) corresponds to baseline peak expiratory flow before running in children. The coefficients (β) and their 95% confidence interval are obtained using linear mixed models. Variables such as sex, age, body mass index, premature birth, rainy season, temperature and relative humidity are forced in all models. Other confounding factors are identified and included: asthma, atopy and Pointoise. The asthma pollution interaction was tested in each model but was not statistically significant.

Abbreviations: PEF, peak expiratory flow; O_3,_ ozone; NO_2_, nitrogen dioxide; CI, confidence interval.

**Table 4:** **Results of linear mixed models to investigate associations between medium-term exposure to indoor and outdoor close-proximity air pollution and peak expiratory flow variation (n = 938)**

	**Indoor**				**Outdoor**			
	**O**_**3**_ **(in μg/m** ^**3**^ **)**		**NO**_**2**_ **(in μg/m** ^**3**^ **)**		**O**_**3**_ **(in μg/m** ^**3**^ **)**		**NO**_**2**_ **(in μg/m** ^**3**^ **)**	
	**β**	**(95% CI)**	**β**	**(95% CI)**	**β**	**(95% CI)**	**β**	**(95% CI)**
Pollutant	0.02	(−0.05; 0.09)	0.00	(−0.16; 0.17)	0.04	(−0.02; 0.10)	0.03	(−0.18; 0.24)
Sex (male vs girl)	−0.06	(−1.35; 1.24)	−0.04	(−1.33; 1.26)	−0.09	(−1.38; 1.21)	−0.03	(−1.33; 1.26)
Age	−0.21	(−1.11; 0.69)	−0.19	(−1.08; 0.71)	−0.22	(−1.11; 0.67)	−0.19	(−1.08; 0.71)
BMI								
*High vs Normal*	0.11	(−1.46; 1.68)	0.14	(−1.43; 1.70)	0.11	(−1.45; 1.67)	0.14	(−1.43; 1.70)
*Low vs Normal*	0.43	(−2.20; 3.07)	0.40	(−2.23; 3.04)	0.48	(−2.15; 3.11)	0.39	(−2.24; 3.02)
Premature birth (Yes vs No)	0.49	(−0.99; 1.97)	0.49	(−0.99; 1.97)	0.48	(−1.00; 1.96)	0.49	(−0.99; 1.97)
Rainy season (Yes vs No)	−0.24	(−1.99; 1.51)	−0.23	(−2.09; 1.62)	−0.42	(−2.08; 1.24)	−0.29	(−2.12; 1.53)
Outside temperature (unit: °C)	0.19	(−0.23; 0.62)	0.16	(−0.25; 0.58)	0.21	(−0.20; 0.61)	0.16	(−0.25; 0.58)
Relative humidity (unit: %)	−0.06	(−0.12; 0.01)	**−0.06**	**(−0.12; 0.00)**	−0.04	(−0.11; 0.02)	**−0.06**	**(−0.12; 0.00)**
Atopy (Yes vs No)	1.09	(−0.38; 2.57)	1.09	(−0.38; 2.57)	1.09	(−0.38; 2.57)	7.52	(−0.39; 2.56)
Variability of school random intercept: ${\widehat{\upsigma}}_{\upgamma 0}^2$ (*P* value)	0.99 (0.10)		1.16 (0.07)		0.6 (0.22)		1.13 (0.08)	

Note. The peak expiratory flow (PEF) corresponds to baseline peak expiratory flow before running in children. The peak expiratory flow variation (ΔPEF) represents the percentage decrease in PEF after running compared to the retained baseline PEF. The coefficients (β) and their 95% confidence interval are obtained using linear mixed models. The variables such as sex, age, body mass index, premature birth, rainy season, temperature and relative humidity are forced in all models. Atopy are identified as confounding factor and included. The asthma pollution interaction was tested in each model but was not statistically significant.

Abbreviations: PEF, peak expiratory flow; O_3,_ ozone; NO_2_, nitrogen dioxide; CI, confidence interval.

With respect to PEF health outcome, there was a statically significant association between the average concentration of outdoor close-proximity pollution by O_3_ and PEF decrease, i.e. a 1-μg/m^3^ increase in O_3_ was associated with a reduction in PEF of 0.32 L/min ($\widehat{\beta}$ = − 0.32; 95% CI: −0.61;-0.03). Conversely, no reduction in PEF was identified with indoor close-proximity pollution by O_3_. NO_2_ was not significantly associated with PEF.

There was no association between ΔPEF and pollutants.

### Association between short- and medium-term exposure to background pollution and health outcomes

Tables 5 and 6 present three models measuring medium-term (model 1 and 2) and short-term (model 3) effects of background pollution on PEF and ΔPEF outcomes in 7 schools.

**Table 5:** **Results of linear mixed models with random intercept on schools to investigate associations between short and medium term background air pollution and peak expiratory flow (n = 425)**

	**O**_**3**_ **(in μg/m** ^**3**^ **)**		**NO**_**2**_ **(in μg/m** ^**3**^ **)**		**SO**_**2**_ **(in μg/m** ^**3**^ **)**		**PM**_**10**_ **(in μg/m** ^**3**^ **)**	
	**β**	**(95% CI)**	**β**	**(95% CI)**	**β**	**(95% CI)**	**β**	**(95% CI)**
**Medium-term exposure**								
**Model 1**								
Pollutant	−0.44	(−2.61; 1.74)	−0.215	(−3.89; 3.46)	1.18	(−2.65; 5.00)	**3.05**	**(0.04; 6.05)**
Variability of school random intercept: ${\widehat{\upsigma}}_{\upgamma 0}^2$ (*P* value)	**90.20 (0.05)**		105.34 (0.11)		**86.80 (0.04)**		11.05 (0.50)	
**Model 2** (Interaction of pollutant with asthma)								
Non-asthmatic children	**−0.13**	**(−2.32; 2.07)**	−0.56	(−4.27; 3.15)	0.67	(−3.20; 4.54)	2.95	(0.12; 5.78)
Asthmatic children	**−1.88**	**(−4.50; 0.74)**	1.40	(−2.97; 5.78)	3.54	(−1.06; 8.14)	3.48	(0.31; 6.64)
Variability of school random intercept: ${\widehat{\upsigma}}_{\upgamma 0}^2$ (*P* value)	**89.30 (0.04)**		107.99 (0.19)		**91.89 (0.04)**		19.82 (0.50)	
**Short-term exposure**								
**Model 3**								
*D0*	−0.24	(−5.09; 4.61)	0.54	(−2.44; 3.51)	0.41	(−35.98; 36.80)	0.14	(−1.82; 2.09)
*D1*	−0.52	(−1.62; 0.58)	0.11	(−2.19; 2.42)	−0.53	(−14.32; 13.27)	0.55	(−1.94; 3.04)
*D2*	−0.56	(−4.06; 2.94)	−0.15	(−2.54; 2.24)	−1.03	(−4.21; 2.14)	0.79	(−2.17; 3.75)
*D3*	−0.37	(−3.68; 2.94)	−0.27	(−2.68; 2.14)	−1.11	(−14.25; 12.04)	0.87	(−2.19; 3.94)
*D4*	0.05	(−0.63; 0.73)	−0.23	(−2.60; 2.15)	−0.74	(−18.06; 16.56)	0.79	(−2.02; 3.59)
*D5*	0.70	(−4.99; 6.39)	−0.03	(−3.06; 2.99)	0.04	(−15.52; 15.60)	0.53	(−1.86; 2.92)
*Cumulative effect*	−0.94	(−4.82; 2.95)	−0.03	(−14.01; 13.94)	−2.96	(−12.69; 6.77)	3.67	(−11.47; 18.81)
Variability of school random intercept ${\widehat{\upsigma}}_{\upgamma 0}^2$ (*P* value)	0.00 (0.50)		0.00 (0.50)		0.00 (0.50)		0.00 (0.50)	

Note. The peak expiratory flow (PEF) corresponds to baseline peak expiratory flow before running in children. The coefficients (β) and their 95% confidence interval were obtained using linear mixed models regarding models 1–2 and a distributed-lag model regarding model 3. Variables such as sex, age, body mass index, full-term birth, rainy season, temperature and relative humidity are forced in each model. Other confounding factors are included: atopy for model 1–2 and atopy, asthma and day of week for model 3.

Short-term exposure was defined by the average of each pollutant concentration on the current day and up to five days corresponding to five different lags (D0, D1, D2, D3, D4, D5). These lags were classified into two categories: short-delay exposure and cumulative short delay exposure, which indicates the mean exposure to pollutants on the five preceding days.

Abbreviations: PEF, peak expiratory flow; O_3,_ ozone; NO2_,_ nitrogen dioxide; SO2_,_ sulphur dioxide; PM10, small particulate matter; CI, confidence interval.

**Table 6:** **Results of linear mixed models with random intercept on schools to investigate associations between short- and medium-term background air pollution and peak expiratory flow variation (n = 355)**

	**O**_**3**_ **(in μg/m** ^**3**^ **)**		**NO**_**2**_ **(in μg/m** ^**3**^ **)**		**SO**_**2**_ **(in μg/m** ^**3**^ **)**		**PM**_**10**_ **(in μg/m** ^**3**^ **)**	
	**β**	**(95% CI)**	**β**	**(95% CI)**	**β**	**(95% CI)**	**β**	**(95% CI)**
**Medium-term exposure**								
**Model 1**								
Pollutant	−0.12	(−0.73; 0.49)	0.67	(−0.01; 1.35)	0.14	(−0.94; 1.22)	**−1.16**	**(−1,95; −0.37)**
Variability of school random intercept: ${\widehat{\upsigma}}_{\upgamma 0}^2$ (*P* value)	6.42 (0.04)		0.93 (0.38)		**6.69 (0.04)**		0.00 (NS)	
**Model 2** (Interaction of pollutant with asthma)								
Non-asthmatic children	−0.14	(−0.76; −0.48)	0.66	(−1.04; 2.37)	0.17	(−0.91; 1.25)	−1.13	(0.16; −2.42)
Asthmatic children	0.00	(−0.74; 0.74)	0.65	(−0.34; 1.64)	−0.06	(−1.37; 1.25)	−1.28	(−0.36; −2.20)
Variability of school random intercept: ${\widehat{\upsigma}}_{\upgamma 0}^2$ (*P* value)	**6.34 (0.05)**		0.97 (0.50)		**6.63 (0.04)**		0.00 (0.50)	
**Short-term exposure**								
**Model 3**								
*D0*	−0.24	(−5.09; 4.61)	0.54	(−2.44; 3.51)	0.41	(−35.98; 36.80)	0.14	(−1.82; 2.09)
*D1*	−0.52	(−1.62; 0.58)	0.11	(−2.19; 2.42)	−0.53	(−14.32; 13.27)	0.55	(−1.94; 3.04)
*D2*	−0.56	(−4.06; 2.94)	−0.15	(−2.54; 2.24)	−1.03	(−4.21; 2.14)	0.79	(−2.17; 3.75)
*D3*	−0.37	(−3.68; 2.94)	−0.27	(−2.68; 2.14)	−1.11	(−14.25; 12.04)	0.87	(−2.19; 3.94)
*D4*	0.05	(−0.63; 0.73)	−0.23	(−2.60; 2.15)	−0.74	(−18.06; 16.56)	0.79	(−2.02; 3.59)
*D5*	0.70	(−4.99; 6.39)	−0.03	(−3.06; 2.99)	0.04	(−15.52; 15.60)	0.53	(−1.86; 2.92)
*Cumulative effect*	−0.94	(−4.82; 2.95)	−0.03	(−14.01; 13.94)	−2.96	(−12.69; 6.77)	3.67	(−11.47; 18.81)
Variability of school random intercept ${\widehat{\upsigma}}_{\upgamma 0}^2$ (*P* value)	0.00 (NS)		0.00 (NS)		0.00 (NS)		0.00 (NS)	

Note. The peak expiratory flow (PEF) corresponds to baseline peak expiratory flow before running in children. The peak expiratory flow variation (ΔPEF) represents the percentage decrease in PEF after running compared to the baseline PEF. The coefficients (β) and their 95% confidence interval were obtained using linear mixed models regarding models 1–2 and a distributed-lag model regarding model 3. Variables such as sex, age, body mass index, full-term birth, rainy season, temperature and relative humidity are forced in each model. Other confounding factors are included: atopy for model 1–2 and atopy, asthma and day of week for model 3.

Short-term exposure was defined by the average of each pollutant concentration on the current day and up to five days before corresponding to five different lags (D0, D1, D2, D3, D4, D5). These lags were classified into two categories: short-delay exposure and cumulative short-delay exposure, which indicates the mean exposure to pollutants on the five preceding days.

Abbreviations: PEF, peak expiratory flow; O3, ozone; NO2, nitrogen dioxide; SO2, sulphur dioxide; PM10, small particulate matter; CI, confidence interval.

In model 1, no significant association was found with O_3_, SO_2_ and NO_2_. However, an interaction within the limits of significance was found between O_3_ and asthma (model 2). This interaction meant that the effect of medium-term exposure to background pollution by O_3_ and NO_2_ on a PEF reduction was stronger in asthmatic children than in non-asthmatic ones. With regard to PM_10_, a significant association with PEF increase and ΔPEF increase (negative variation) was found (Tables 5 and 6).

In model 3, short delay (D0, D1, D2, D3, D4, D5) exposure and cumulative short delay exposure were not associated with PEF and ΔPEF.

## Discussion

The asthma prevalence identified in this study was slightly higher than that reported in the few studies conducted in the Caribbean using comparable standardized ISAAC methodology [18,20]. The previous Guadeloupian study reported a prevalence of 14.1% among 5,094 adolescents with a mean age of 12.9 years and another study in the Caribbean islands of Trinidad and Tobago reported 12.8% and 13.5% respectively, among 4,988 adolescents. Although the present study concerned younger children (a mean age of 10.3 among 1,436 children), our findings confirm the high asthma prevalence in the Caribbean compared with the prevalence worldwide [28]. Furthermore, the studies conducted in the Caribbean suggest small variations in asthma prevalence between the Caribbean islands and contrast with a recent study conducted in the Pacific where considerable variations were found, the rates ranging from 5.8% in Samoa to 19.7% in the Tokelau Islands [29].

Many factors influence asthma such as genetic susceptibility and cultural differences [30]. A previous ISAAC phase two study conducted in Hong Kong and two cities in mainland China showed that the higher prevalence of asthma symptoms in Hong Kong could be explained by differences in environmental factors and diet [31]. The impact of environmental factors on respiratory diseases, particularly air pollution, seems to vary in different parts of the globe, probably owing to individual susceptibility.

To our knowledge, this study is the first to provide close-proximity and background pollution data in the Caribbean and to measure its impact on the respiratory lung function of elementary schoolchildren. The observed mean concentrations of PM_10_ in 5 out of the 7 participating schools exceed the known threshold limit of 20 μg/m^3^ proposed by WHO air quality guidelines [32]. A recent French ministry report explained that the increase in PM_10_ concentration was partly due to Saharan-Sahel dust carried by atmospheric circulation over the Caribbean area for five to seven months a year [33]. In contrast with PM_10_, the observed mean concentrations of O_3,_ NO_2_ and SO_2_ were lower than the WHO known threshold limit values of 100 μg/m^3^ for O_3_, 40 μg/m^3^ for NO_2_, and 20 μg/m^3^ for SO_2_. This low air pollution level is not striking with respect to the regional emission density of SO_2_ and NO_2_ in the Caribbean, which is still far less than that of many temperate-zone industrial countries. However for O_3_, which is an atmospheric pollutant not directly emitted by car engines or by industrial operations but formed by the reaction of sunlight on air containing hydrocarbons and nitrogen oxides that react to form O_3,_ the relatively low concentration was not expected in Guadeloupe where temperature and sunlight intensity are high throughout the year. The impact of wind could explain these variations. In addition, another source of NO2 and SO2 emissions could be wood-burning in rural areas and fires in sugar cane plantations before harvesting begins.

The main strength of this study is that it assessed medium-term exposure to both indoor and outdoor close-proximity air pollution. Indeed, elementary schoolchildren spend a lot of time at school in many countries and almost 8 hours per day in an academic year in France. For this reason, this study provides an interesting insight into the effect of air pollution in schools. Medium-term exposure to both indoor and outdoor close-proximity air pollution was assessed by measuring the two-week cumulative effect of two major pollutants such as O_3_ and NO_2_ in a large random sample of Guadeloupian classrooms. Even with levels lower than WHO guidelines, the linear mixed model analyses showed significantly reduced lung function before exercise with outdoor exposure to O_3_ after controlling for personal attributes and meteorological factors.

Most studies in schoolchildren have already provided evidence that exposure to O_3_ reduces lung function after controlling for confounders but not at such a low level [34]. To our knowledge, only a recent study suggested that exposure to similar low levels of O_3_ could have an impact on asthma. The authors reported an association between emergency pediatric department visits for asthma and relatively low O_3_ concentrations [35]. Moreover, we showed a stronger association of medium-term exposure to background pollution by O_3_ with PEF reduction in asthmatic children than in non-asthmatic children. This seems plausible because air pollution may act not only as a short-term trigger but also as a priming event inducing mechanisms of enhanced airway inflammation, which in turn result in subsequent bronchial hyperactivity [36].

Although our results concerning medium-term exposure to indoor pollutants suggest no meaningful association with a PEF, several studies have identified associations between air pollution in classrooms and the respiratory health of schoolchildren [37,38]. For instance, a recent French study assessing several pollutants (PM_2.5_, NO_2_ and three aldehydes) showed that poor air quality in classrooms was related to an increased prevalence of clinical manifestations of asthma and rhinitis [37].

The relatively low concentrations of SO2 and NO2 compared to those of quality guidelines could explain why no association was identified with a decrease in lung function. Indeed, we observed a factor close to 2 between air quality guidelines and the concentration mean of O_3_ and a factor close to 3 and 4 with NO_2_ and SO_2_ respectively. With respect to PM_10_, we found significant associations with lung function that increased for PEF and ΔPEF. This result is really puzzling because it is difficult to imagine that exposure to PM_10_ has a protective effect. A possible explanation is that we performed a total of 24 analyses and observed significant associations with outcomes just by chance. However, our results on O_3_ point to a significant association not due to chance since O_3_ associations were found with decreasing PEF both for medium-term exposure to outdoor close-proximity pollution and for background pollution (interaction with asthma). In addition, the use of passive diffusion samplers to measure pollutant concentrations in the schools is more reliable than the method using fixed stations, which underlines the pertinence of our findings about O3 regarding medium-term exposure to outdoor close-proximity pollution.

Our study has some limitations. First, lung function was measured with a peak flow meter instead of spirometry, which provides more lung function parameters. However, peak flow meters are inexpensive, portable, easy to use and do not need to be plugged in. Moreover, PEF is still used in practice for asthma monitoring according to the guidelines in the Global Initiative for Asthma report [39] in epidemiological studies with trained technicians [40] and in clinical trials [41].

A second limitation is that the analyses of short- and medium-term exposure to background pollution were based on a small sample of 7 schools that did not reflect spatial variations. In addition, the lack of power could lead to a wide confidence interval and no significant association in the analyses. However, we were unable to collect data in the other participating schools because the fixed stations were located only in the agglomeration of Pointe-à-Pitre. With respect to close-proximity air pollution, a large sample size was used as recommended in the ISAAC 2 protocol, so robust analyses were possible.

The confounding role of medications for asthma may be considered as another potential limitation. Medication use in children may influence lung function and mask the effects of pollutants. For instance, asthmatic children taking a corticoid treatment on the days preceding the run test will be less susceptible to air pollutants than other children. In addition, the school environment may not have been the only factor responsible for decreasing lung function because some children may have been exposed to air pollution at home.

As in most studies of this type, unmeasured confounding is a real concern. Socio-economic factors may correlate with spatial variation in air pollution. Indeed, lower socio-economic groups are likely more exposed and more susceptible to air pollution. For this reason, they might have an effect on the parameters we measured. An analysis including socio-economic factors could highlight air pollution effects in lower socio-economic groups which could not be detected in this study.

A final limitation was the lack of PM2.5 measurements, as they were not being monitored by the GWAD’AIR Association when the study started.

## Conclusions

This observational exploratory study provided the opportunity to measure the impact of exposure to low concentration air pollution on lung function among a population of children with a high prevalence of asthma. It bridges the knowledge gap regarding pollution in the Caribbean and demonstrates a consistent association between medium-term exposure to low O_3_ concentrations under the WHO threshold levels and reduced lung function in children. However, additional research in the Caribbean is needed to confirm our results and to study the impact of other pollutants. For instance, our study was limited to measuring two indoor pollutants owing to logistical difficulties and economic constraints. In addition, further research is needed to measure the toxicological and biological effects of particles originating from Saharan-Sahel dust, as suggested in another study [42]. In the future, new statistical approaches combining pollutant mixtures will be useful to better understand the role of air pollutant concentrations on the development of asthma and allergies in the Caribbean [43].

## Abbreviations

BMI: Body mass index
CI: Confidence interval
NO_2_: Nitrogen dioxide
O_3_: Ozone
PEF: Peak expiratory flow
PM_10_: Particles with aerodynamic diameter lower than 10 μm
SD: Standard deviation
SO_2_: Sulfur dioxide
